# Transcriptome-Based Identification of Novel Transcription Factors Regulating Seed Storage Proteins in Rice

**DOI:** 10.3390/plants14172791

**Published:** 2025-09-05

**Authors:** Jinpyo So, Jong-Yeol Lee, Kyoungwon Cho, Suchan Park, Kyuhee Lee, Don-Kyu Kim, Oksoo Han

**Affiliations:** 1 Department of Biotechnology and Department of Integrative Food, Bioscience and Biotechnology (BK21 FOUR), Chonnam National University, Gwangju 61186, Republic of Korea; thwlsvy123@gmail.com (J.S.); kw.cho253@gmail.com (K.C.); heumny@naver.com (S.P.); leekh@maeil.com (K.L.); dkkim2@jnu.ac.kr (D.-K.K.); 2 Department of Agricultural Biotechnology, National Institute of Agricultural Science, RDA, Jeonju 54874, Republic of Korea; jy0820@korea.kr

**Keywords:** seed storage protein, transcription factor, glutelin, prolamin, seed quality, transcriptome analysis

## Abstract

Seed storage proteins (SSPs) play a pivotal role in determining the development, quality, and nutritional value of rice seeds. In this study, we conducted a transcriptome-based correlation analysis to identify novel transcription factors (TFs) potentially involved in the biosynthesis and accumulation of SSPs. Our analysis revealed nine TFs—OsGATA8, OsMIF1, OsMIF2, OsGZF1, OsbZIP58, OsS1Fa1, OsS1Fa2, OsICE2, and OsMYB24—that exhibit strong co-expression with key SSP genes, including those encoding glutelin and prolamin. Gene expression profiling using quantitative RT-PCR and GUS reporter assays revealed that these TFs are predominantly expressed during seed development, with peak expression observed at 10 days after flowering (DAF). Promoter analysis further demonstrated an enrichment of seed-specific and hormone-responsive cis-regulatory elements, reinforcing the seed-preferential expression patterns of these TFs. Collectively, our findings identify a set of candidate TFs likely involved in SSP regulation and seed maturation, providing a foundation for the genetic enhancement of rice seed quality and nutritional content through targeted breeding and biotechnological approaches.

## 1. Introduction

Rice (*Oryza sativa* L.), along with wheat and maize, is among the most extensively cultivated and consumed cereal crops worldwide, serving as a staple food particularly in Asia. It possesses agronomic and nutritional traits critical for both cultivation and human consumption, including yield, grain quality, stress resilience, nutritional composition, and growth duration. Of these, grain quality and nutritional value are primarily dictated by the composition and relative abundance of nutrients in the rice seed [1,2].

Rice seeds serve as a major source of nutrients for humans, predominantly providing energy in the form of starch, along with proteins, lipids, vitamins, and minerals. On a dry weight basis, rice seeds comprise approximately 80% carbohydrates, 8–10% protein, less than 1% lipid, and about 3.5% dietary fiber [2,3]. Seed storage proteins (SSPs), which account for the largest proportion of total protein in rice seeds, are classified into four types based on their solubility: glutelin, prolamin, globulin, and albumin. Among these, glutelin is the most abundant (60–80%), followed by prolamin (20–30%), globulin (~5%), and albumin (~10%) [4,5,6].

SSPs contribute significantly to seed size and weight, and are integral to seed development, nutrient storage, and maturation [1,7]. RNA interference-mediated suppression of glutelin, prolamin, and globulin genes in rice has revealed corresponding reductions in grain weight and starch content, along with altered aleurone and endosperm structures and delayed germination [8]. The knockout of glutelin genes using CRISPR-Cas9 has been shown to produce similar phenotypes, including a reduction in seed weight, morphological changes, and disrupted endosperm structure with lower levels of starch and SSPs [9,10,11]. Furthermore, editing of 13 kDa prolamin genes using CRISPR-Cas9 induced compensatory changes in the expression of other SSPs, with concomitant effects on starch composition and seed structure [12].

The expression and deposition of SSPs are regulated by environmental conditions (e.g., temperature, humidity, nutrient availability) and intrinsic factors such as transcription factors (TFs) and endogenous phytohormones [2,13,14,15]. Several TFs have been identified that modulate SSP expression. For instance, NF-YC12, a component of the NF-Y TF complex, is crucial for starch and SSP accumulation. Loss of its function reduces grain weight and disturbs nutrient deposition [14]. RISBZ1, a type of the basic leucine zipper (bZIP), binds the GCN4 motif to activate endosperm-specific expression of SSP genes and interacts with the rice prolamin box-binding factor (RPBF) to regulate SSP biosynthesis and seed development [16,17]. Conversely, OsGZF1, a CCCH-type zinc finger protein, suppresses *GluB-1* expression by inhibiting RISBZ1-mediated activation [18]. OsNAC20 and OsNAC26, members of the NAC TF family, are expressed in the embryo and endosperm and directly enhance transcription of genes involved in starch and SSP biosynthesis. CRISPR-Cas9-mediated knockout of OsNAC20 and OsNAC26 led to a marked decrease in both starch and SSP content [19].

These findings collectively suggest that SSP biosynthesis is intricately coordinated with starch metabolism and seed developmental processes. Notably, mutations in TFs associated with SSP regulation consistently manifest in altered grain morphology and compositional shifts [14,16]. Identification of novel TFs involved in SSP regulation is therefore pivotal for understanding seed development and improving the nutritional quality of rice.

In this study, transcriptomic datasets [20] were employed to identify transcription factors that may potentially be involved in the regulation of seed storage protein expression. Based on these datasets, we constructed a co-expression network to comprehensively examine the relationships between transcription factors and SSP-related genes. Through this network analysis, novel transcription factors closely associated with SSP expression in rice were systematically identified. To support these findings, we validated the expression patterns of the candidate TFs, thereby providing multiple lines of evidence for their potential involvement in SSP regulation.

## 2. Results

### 2.1. Identification of Novel Transcription Factors Correlated with Seed Storage Protein Genes

To identify transcription factors (TFs) potentially involved in the regulation of rice seed storage proteins (SSPs), we constructed a co-expression network based on the rice seed transcriptome derived from the Agilent 4 × 44 K custom oligo-DNA chip (Agilent Technologies, Santa Clara, CA, USA). Annotation of genes using the MapCave tool identified 38 SSP-encoding genes on the array, including 25 prolamins, 11 glutelins, one globulin, and one albumin (Table S1). From a previous RNA-seq dataset of immature seeds from SSP-deficient lines [20], 128 TFs among the top 2000 most abundant transcripts were selected based on functional annotation.

**Figure 1:**
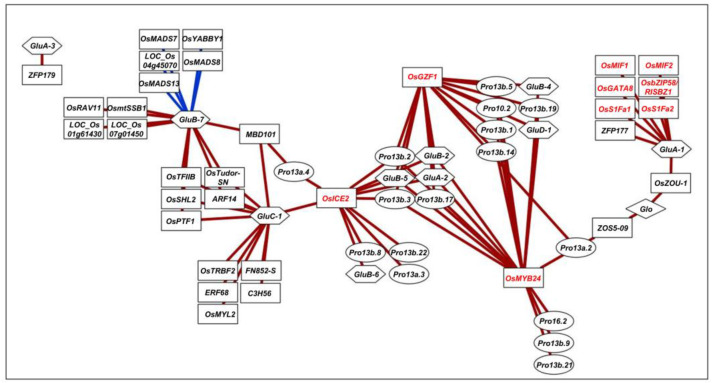
Co-expression network between transcription factor- and seed storage protein-related genes. The hexagonal boxes represent glutelin proteins, the circular boxes represent prolamin proteins, and the square boxes represent transcription factors. The red lines indicate positive correlation coefficiency, while the blue lines indicate negative correlation coefficiency. The proteins highlighted in red are the selected transcription factors.

Pearson’s correlation analysis of gene expression profiles across 48 transcriptome datasets revealed strong associations (|r| ≥ 0.7) between 27 SSP and 33 TF genes (Table S1). These interactions were visualized as a co-expression network using Cytoscape (Figure 1). Notably, three TFs—*OsMYB24*, *OsGZF1*, and *OsICE2*—emerged as hubs, correlating with 17, 14, and 12 SSP genes, respectively. These hub TFs shared co-expression with six SSP genes: *GluA-2*, *GluB-2*, *GluB-5*, *Pro13b.2*, *Pro13b.3*, and *Pro13b.17*. Most TFs showed correlation with only a single SSP gene (e.g., nine TFs with *GluB-7*, five with *GluC-1*, seven with *GluA-1*, and one with *GluA-3*).

**Figure 2:**
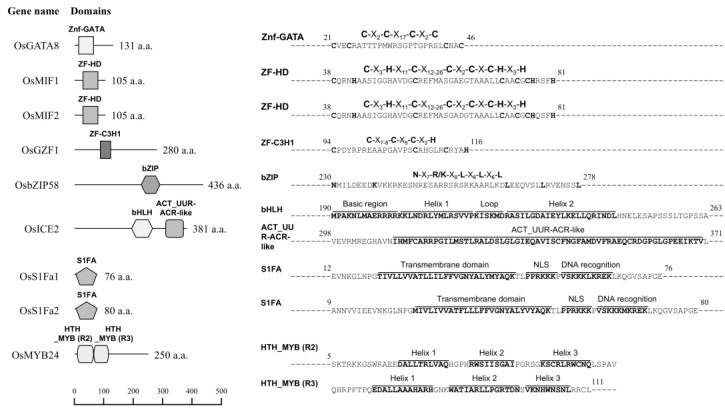
Schematic representation of the domain predicted to be present in selected TF proteins.

Among these, we focused on nine TFs that either functioned as network hubs or were linked to *GluA-1*, whose functional disruption via CRISPR-Cas9 affects grain traits [10]. These included *OsGATA8* (GATA transcription factor 8), *OsMIF1* (mini zinc finger 1), *OsMIF2* (mini zinc finger 2), *OsGZF1* (GluB-1-binding Zinc Finger 1), *OsbZIP58* (bZIP transcription factor 58), *OsS1Fa1* (site 1 binding factor a1), *OsS1Fa2* (site 1 binding factor a2), *OsICE2* (Inducer of CBF expression 2) and *OsMYB24* (R2R3-MYB transcription factor 24) (Figure 2).

To further validate the regulatory potential of the candidate TFs identified through the co-expression network, we performed a transient overexpression assay in rice protoplasts. The TFs analyzed were OsGATA8, OsMIF1, OsbZIP58, and OsS1Fa1, which are co-expressed with *GluA-1*. Compared with the control, the expression levels of SSP genes were generally increased in protoplasts overexpressing the TFs. In particular, OsbZIP58 markedly enhanced the expression of genes of *GluA* group and also led to a pronounced up-regulation of most prolamin genes (except *Pro13a-1* and the *Pro16* group) as well as the globulin gene (Figure S1). These results confirm that the selected TFs can functionally contribute to the regulation of SSP gene expression.

### 2.2. Protein–Protein Interaction (PPI) Network Analysis of Candidate TFs

The nine candidate TFs were classified into four families based on Rice Genome Annotation Project, RAP-DB, and InterPro data (Table 1): zinc finger (C3H1: OsGZF1; GATA: OsGATA8; ZF-HD: OsMIF1, OsMIF2), basic domain (bZIP: OsbZIP58; bHLH: OsICE2), S1Fa (OsS1Fa1, OsS1Fa2), and MYB (R2R3: OsMYB24).

**Figure 3:**
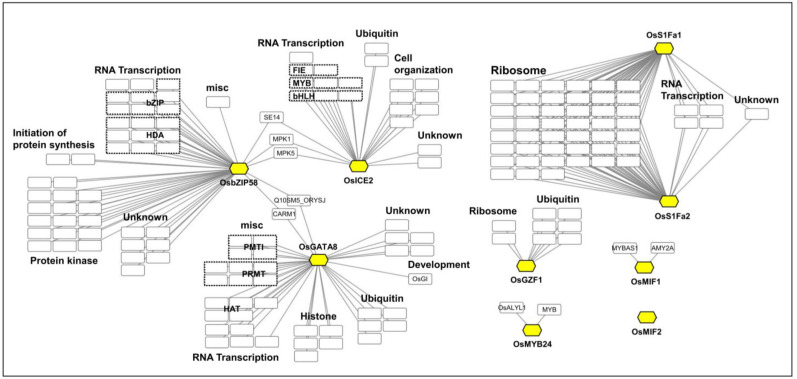
Protein–protein interaction network of each TF. The hexagonal boxes filled with yellow represent the selected TF proteins, and the square-shaped boxes represent proteins predicted to interact with the TF.

PPI predictions using STRING (confidence score ≥ 0.4) revealed diverse molecular interactions (Figure 3). OsbZIP58 interacted with proteins involved in RNA transcription, initiation of protein synthesis, protein kinase, misc, and unknown functions. OsICE2 was associated with components of RNA transcription, ubiquitin, cell organization, and unknown functions. OsGATA8 interacted with proteins linked to RNA transcription, miscellaneous functions, histone, ubiquitin, development, and unknown functions. OsS1Fa1 and OsS1Fa2 were associated with ribosome, RNA transcription, and unknown functions. OsGZF1 interacted with proteins involved in ubiquitin and ribosome. OsMIF1 showed interactions with MYBAS1 and AMY2A, while OsMYB24 interacted with OsALYL1 and MYB-related proteins. No interacting partners were identified for OsMIF2.

Notably, no direct interactions were observed among the nine TFs, consistent with results from the yeast two-hybrid (Y2H) assay (Figure S2). However, OsICE2 and OsbZIP58 commonly interacted with SE14, MPK1, and MPK5, while OsbZIP58 and OsGATA8 shared interactions with CARM1 and Q10SM5_ORYSJ.

### 2.3. Expression Profiles of TFs During Seed Development

**Figure 4:**
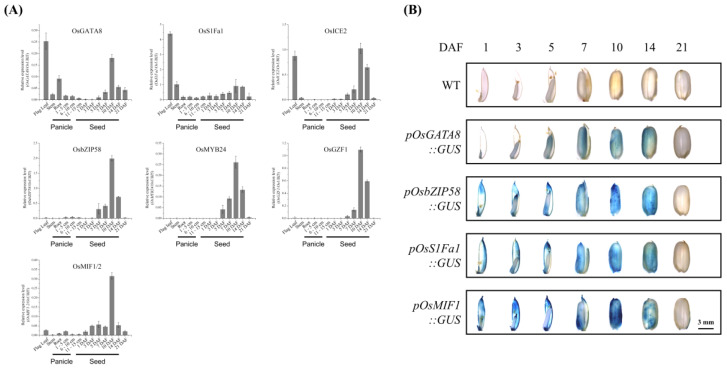
Expression patterns of TFs in different tissues and stages. (**A**) qRT-PCR analysis of TFs. The expression of TFs was normalized to that of *OsUBI5*. (**B**) Histochemical assay of GUS activity in developing seeds of transgenic rice plants expressing four TF promoter-GUS fusion constructs. Rice seeds were collected 1–21 days after flowering (DAF).

qRT-PCR analysis of TF gene expression across reproductive tissues revealed two major expression patterns (Figure 4A). One group, consisting of *OsbZIP58*, *OsGZF1*, *OsMYB24*, and *OsMIF1/2*, displayed seed-specific expression peaking at 10 days after flowering (DAF), followed by a decline. The other group, including *OsGATA8*, *OsS1Fa1*, and *OsICE2*, exhibited both seed-specific and leaf-enriched expression profiles.

To further investigate temporal and spatial expression patterns during seed development, four proTF::GUS transgenic lines were generated using 1.5 kb upstream promoter sequences from *OsGATA8*, *OsS1Fa1*, *OsbZIP58*, and *OsMIF1* (Figure 4B and Figure S3). GUS staining revealed consistent expression across lines, initially localized to the seed tips until 5 DAF, expanding throughout the seed during development, peaking at 10 DAF, and diminishing as seeds matured and hardened.

### 2.4. Genome-Wide Enrichment Analysis of Cis-Regulatory Elements (CREs)

To explore regulatory motifs associated with TF expression, genome-wide CRE enrichment analysis was conducted on the 2000 bp upstream promoter regions of the nine candidate TFs using the PLACE database and MEME Suite (Figure S4, Table S5). Ten CREs were significantly enriched (*p* ≤ 0.1) in five TF promoters: *OsMYB24*, *OsbZIP58*, *OsGATA8*, *OsICE2*, and *OsGZF1*.

**Figure 5:**
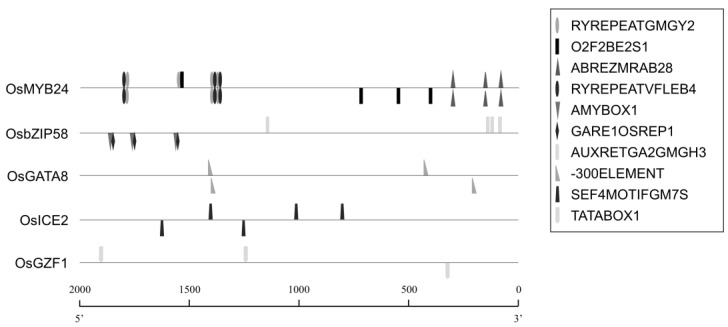
Distribution of significantly enriched cis-regulatory elements (*p* ≤ 0.1) in the 2 kb upstream promoter regions of TF genes.

Specifically, *OsMYB24* contained four enriched motifs (ABREZMRAB28, O2F2BE2S1, RYREPEATGMGY2, RYREPEATVFLEB4), *OsbZIP58* had three (AMYBOX1, GARE1OSREP1, AUXRETGA2GMGH3), *OsGATA8* one (-300ELEMENT), *OsGZF1* one (TATABOX1), and *OsICE2* one (SEF4MOTIFGM7S) (Figure 5, Table 2). No significant CRE enrichment was observed in *OsMIF1*, *OsMIF2*, *OsS1Fa1*, or *OsS1Fa2* promoters.

## 3. Discussion

Efforts to suppress the synthesis of seed storage proteins (SSPs) in rice—through CRISPR-Cas9-mediated gene editing or transcriptional silencing via RNA interference—have often resulted in compensatory upregulation of non-targeted SSPs, thereby maintaining total seed protein content [12,35,36]. These findings underscore the necessity for a deeper understanding of SSP regulatory networks.

It has been revealed that SSP biosynthesis influences starch metabolism by altering carbon–nitrogen partitioning, modulating the sucrose–hexose balance, and providing metabolic signals that regulate starch biosynthetic pathways [37]. This suggests that a deeper understanding of SSP regulatory networks may not only improve knowledge of protein accumulation but also provide critical insights into the coordinated synthesis and deposition of multiple storage reserves in seeds.

In this study, we conducted a comprehensive co-expression analysis and identified nine transcription factors (TFs)—*OsICE2*, *OsGZF1*, *OsMYB24*, *OsMIF1*, *OsMIF2*, *OsGATA8*, *OsbZIP58*, *OsS1Fa1*, and *OsS1Fa2*—that are strongly associated with SSP gene expression (Figure 1). Expression profiling using qRT-PCR and promoter-driven GUS reporter assay in transgenic rice confirmed that these TFs are highly expressed during seed development (Figure 4). Furthermore, transient overexpression assays in rice protoplasts demonstrated that selected TFs, particularly OsbZIP58, can directly activate multiple SSP genes, providing functional support for our network-based predictions (Figure S1). However, as these assays were limited to a protoplast system, further in planta validation such as stable transformation or mutant analysis will be required to confirm their regulatory roles during seed development.

Among them, OsbZIP58 and OsGZF1 have previously been identified as regulators of seed-specific gene expression [16,17,18]. OsbZIP58, a member of the bZIP family, contains a basic DNA-binding domain and a leucine zipper domain mediating DNA binding and protein–protein interactions, respectively [38]. As a transcriptional activator, it regulates genes involved in starch biosynthesis and SSP accumulation during seed development [39,40]. SSP gene promoters commonly harbor cis-regulatory elements (CREs) such as AACA, ACGT, GCN4, and PROL. OsbZIP58, predominantly expressed in the endosperm and aleurone layer, binds to GCN4, ACGT, and ATGA motifs to activate seed-specific gene expression [17,41,42]. It also interacts synergistically with RPBF (Rice Prolamin Box Binding Factor), a member of the Dof family, to promote storage protein gene expression [16,43].

In this study, the *OsbZIP58* promoter was found to contain the seed-specific motif AMYBOX1 (TAACARA) and the auxin-responsive element AUXRETGA2GMGH3 (TGACGTGGC) (Figure 5, Table 2). Co-expression and yeast two-hybrid (Y2H) analyses showed that OsbZIP58 is closely correlated with GluA-1, but not with other TFs (Figure 1, Figure S2). However, PPI analysis indicated that SE14, MPK1, and MPK5 bind with both OsbZIP58 and OsICE2, and Q10SM5_ORYSJ and CARM1 interact with both OsbZIP58 and OsGATA8, suggesting that the interaction between the TFs is mediated by other proteins (Figure 3). To provide more definitive evidence of these interactions, further experimental validation such as Y2H or BiFC will be necessary.

OsICE2, a TF of the bHLH family that includes a total of 167 genes in rice [44,45], contains a single helix–loop–helix (HLH) motif and an ACT_UUR-ACR-like domain in its C-terminal region (Figure 2). OsICE2 plays an important role in plant growth and development, particularly in response to cold stress and in stomatal development [22,23,46,47]. As shown in Figure 4A, *OsICE2* expression gradually increases until 10 DAF and then declines, exhibiting a pattern similar to that of 12 SSP genes (Figure 1). Promoter analysis further revealed significant enrichment of the SEF4MOTIFGM7S element (RTTTTTR), a known cis-regulatory element associated with seed storage protein gene expression [33].

OsGATA8 is a TF belonging to the GATA-type zinc finger protein family, which recognizes GATA motifs in DNA and plays a crucial role in signaling pathways related to abiotic stresses, including salt, drought, and exogenous abscisic acid (ABA) [48,49]. Previous studies have linked OsGATA8 to water stress responses and tiller formation [21,50]. Co-expression network analysis revealed that *OsGATA8* exhibits a transcriptional pattern similar to that of *GluA-1*, a major SSP gene (Figure 1).

Promoter analysis showed that the *OsGATA8* promoter is significantly enriched in the -300ELEMENT motif (TGHAAARK), an enhancer element identified in glutenin promoters that drives endosperm-specific expression [32]. Furthermore, both the expression profile of *OsGATA8* during seed maturation and GUS reporter activity driven by the *OsGATA8* promoter::GUS transgenic rice supports its involvement in seed development.

OsGZF1 is a TF belonging to the C3H zinc finger protein family, which plays crucial roles in stress responses and developmental processes in roots, leaves, flowers, and seeds [51]. OsGZF1 has been reported to repress the expression of *GluB-1* by binding to AACA, ACGT, GCN4, and PROL motifs within its promoter, thereby interfering with the activation of *GluB-1* by OsbZIP58 [17,41]. In *OsGZF1* RNAi mutants, down-regulation of OsGZF1 led to increased grain nitrogen content, suggesting that OsGZF1 binds to promoters of glutelin genes, including that of *GluB-1*, to repress their expression [18]. In this study, PPI and Y2H analysis showed no direct interaction between OsGZF1 and OsbZIP58 (Figure 3, Figure S2). Considering that both OsbZIP58 and OsGZF1 bind to the same cis-elements on the promoter of SSP genes and OsbZIP58 activates the expression of SSPs but OsGZF1 represses it, the results suggest that OsbZIP58, as an activator, and OsGZF1, as a repressor, might compete to regulate the expression of SSPs. Promoter analysis of *OsGZF1* (Figure 5, Table 2) revealed a significant enrichment of TATABOX1 (CTATAAATAC), a motif highly conserved in the promoters of SSP genes in the Poaceae family [52]. Furthermore, co-expression network analysis showed that *OsGZF1* shares expression patterns with 14 SSP genes, including *GluA-2*, *GluB-2*, *GluB-4*, *GluB-5*, *GluD-1*, *Pro10.2*, *Pro13a.2*, *Pro13b.1*, *Pro13b.2*, *Pro13b.3*, *Pro13b.5*, *Pro13b.14*, *Pro13b.17*, and *Pro13b.19*, whereas *OsbZIP58* is more specifically co-expressed with *GluA-1* (Figure 1). The result suggests that the existence of at least two regulatory modes for controlling SSP content: One is that SSP content could be properly controlled by the co-occurrence of a large number of SSP gene expression activities with *OsGZF1*. The other is that the expression activities of a small number of SSP genes, including *GluA-1*, might occur with that of *OsbZIP58*, activating the expression of a large number of SSP genes.

OsMYB24 is a TF belonging to the R2R3-type MYB family, characterized by two tandem MYB domains (R2 and R3). Each domain consists of a three-helix structure comprising 51 to 53 amino acids (Figure 2), and specifically recognizes the YAAC(G/T)G DNA sequence [53,54], potentially playing a crucial role in gene expression for plant development, cell differentiation, and specialized metabolism such as lignin and flavonoid biosynthesis. During seed development, co-expression analysis (Figure 1) revealed that *OsMYB24* is co-expressed with three prolamin genes (*Pro13b.9*, *Pro13b.21*, and *Pro16.2*) as well as 14 SSP genes that also show co-expression with *OsGZF1*. Promoter analysis of *OsMYB24* showed significant enrichment of three seed-specific CREs—O2F2BE2S1 (GCCACCTCAT), RYREPEATGMGY2 (CATGCAT), and RYREPEATVFLEB4 (CATGCATG)—as well as an ABA/water–stress-responsive CRE, ABREZMRAB28 (CCACGTGG). Expression profiling during seed maturation (Figure 4A) demonstrated that *OsMYB24* exhibits a transcriptional pattern similar to that of *OsGZF1*. These findings suggest a potential role for OsMYB24 in regulating seed storage protein expression, highlighting the need for further functional studies to elucidate its involvement in seed development.

OsS1Fa1 and OsS1Fa2 are small proteins composed of 76 and 80 amino acids, respectively (Figure 2), and belong to the S1Fa TF family, which is characterized by a nuclear localization signal and a putative DNA-binding helix [55,56]. The S1Fa family is known to specifically recognize the negative promoter element S1F, repressing the expression of RPS1, a gene encoding the chloroplast ribosomal protein S1, particularly in non-photosynthetic tissues such as roots. Additionally, S1Fa TFs are highly expressed under drought stress conditions, where they contribute to the suppression of *RPS1* expression as part of the drought response [24,57]. In this study, *OsS1Fa1* showed elevated expression in the rice flag leaf at 21 DAF compared to other tissues, including roots, stems, and seeds. In developing seeds, *OsS1Fa1* expression peaked at 10 DAF and declined thereafter. These results suggest that OsS1Fa1 may be involved in seed developmental regulation, in addition to its known role in stress responses. Moreover, PPI network analysis of OsS1Fa1 and OsS1Fa2 (Figure 2) predicted potential interactions with ribosomal proteins (Figure 3, Tables S3 and S4), implying a possible role in the post-translational regulation of ribosome biogenesis.

OsMIF1 and OsMIF2 are homeodomain-deficient TFs that belong to the plant-specific ZF-HD TF family, which is known to play critical roles in environmental stress responses, as well as the growth and development of floral and seed structures [58,59]. In rice, the ZF-HD family comprises 11 canonical ZF-HD members, each containing both a C5H3-type zinc finger domain and a homeodomain, and 4 MIFs (mini zinc finger proteins, OsMIF1–4), which lack the homeodomain. Although the roles of OsMIF1 and OsMIF2 in rice are currently unclear, their homologs in other species, AtMIF2 in Arabidopsis, SlIMA in tomato, and GhMIF in Gerbera, have been reported to be involved in floral meristem termination, floral development, and ovule development [60,61]. In this study, GUS reporter assays driven by the *OsMIF1* promoter and qRT-PCR analysis of *OsMIF1* and *OsMIF2* expression (Figure 4) suggest that both TFs may play a role in the reproductive stage of rice development.

Our results provided important clues for identifying candidate TFs involved in the regulation of SSPs. These approaches open the possibility of uncovering novel regulatory mechanisms of SSP expression that have not been previously reported, thereby offering new directions for future research. In particular, for the TFs whose functions have not yet been characterized, our results indirectly suggest their potential roles in seed development and SSP regulation. Nevertheless, these results do not provide direct evidence that the identified TFs regulate SSP expression. Future functional studies may help to establish their precise contributions to SSP biosynthesis and grain development, thereby validating and extending the hypotheses proposed in this study.

## 4. Materials and Methods

### 4.1. Correlation Analysis of Transcription Factor and Seed Storage Protein Genes Using Rice Seed Transcriptome

The 2000 most abundant transcripts in immature seeds of rice (*Oryza sativa* cv. Ilmi) were selected from previously published RNA-Seq data [20] and annotated using MSU locus identifiers from the Rice Genome Annotation Project (http://rice.plantbiology.msu.edu/, accessed on 7 August 2025). Functional categorization was performed based on reference annotations from the MapManStore database for the *O. sativa* Japonica group (https://mapman.gabipd.org/mapmanstore, accessed on 7 August 2025).

To evaluate gene co-expression between transcription factors (TFs) and seed storage proteins (SSPs)—including glutelins, prolamins, and globulins—Pearson’s correlation coefficients were calculated (cut-off: |r| ≥ 0.7) using Microsoft Excel (Table S1). The rice seed transcriptome was compiled from 46 publicly available microarray datasets (GEO Series: GSE30462, GSE37478, GSE37650, GSE49963, and GSE79405) and 43 in-house microarray experiments based on the Agilent Rice 4 × 44 K platform (Agilent Technologies, Santa Clara, CA, USA). Co-expression networks were visualized using Cytoscape (v3.10.2).

### 4.2. Information on Selected Transcription Factors

Genomic loci and sequences of selected TFs were collected from the Rice Genome Annotation Project (http://rice.uga.edu, accessed on 7 August 2025) and RAP-DB (https://rapdb.dna.affrc.go.jp, accessed on 7 August 2025). Functional annotations were based on literature cited in Table 1. Protein domains were predicted using InterPro (https://www.ebi.ac.uk/interpro, accessed on 7 August 2025).

### 4.3. Protein–Protein Interaction (PPI) Network Analysis

To infer functions of hub genes co-expressed with SSP genes, PPI networks were constructed using the STRING database (https://string-db.org, accessed on 7 August 2025) with a minimum confidence score of 0.4. Network visualizations were generated in Cytoscape (v3.10.2) based on STRING outputs.

### 4.4. Plant Materials and Growth Conditions

Seeds of *O. sativa* L. ssp. Japonica cv. Ilmi were sourced from the Korea Seed & Variety Service (Gimcheon, Korea). Plants were cultivated in paddy fields under standard agronomic conditions.

### 4.5. Rice Protoplast Transient Expression

Rice protoplasts were isolated from 8–12 days old seedlings by enzymatic digestion (cellulase and macerozyme) and purified using W5/MMG buffers. For transient overexpression, 5–10 µg of plasmid DNA carrying OsGATA8, OsbZIP58, OsS1Fa1, or OsMIF1 was introduced into protoplasts via PEG-mediated transformation, and cells were incubated for 16–24 h at room temperature. Total RNA was extracted from transformed protoplasts.

### 4.6. RNA Extraction and Quantitative RT-PCR

Total RNA was isolated from leaves, roots, and stems at 21 days after flowering (DAF), as well as from immature seeds at various developmental stages, using a modified protocol optimized for starchy tissues [62]. The cDNA library was synthesized by using 1 µg of RNA with QuantiTect Reverse Transcription Kit (Cat. #205311, Qiagen, Hilden, Germany) according to the manufacturer’s protocol. qRT-PCR was conducted on a Rotor-Gene Q (Qiagen) using 10 ng of cDNA, 0.5 µM gene-specific primers, and 10 µL of 2× SYBR Green Master Mix (Qiagen, Cat. #204343, Hilden, Germany) in a 20 µL total volume. Thermal cycling was as follows: 95 °C for 10 min; 40 cycles of 95 °C for 30 s, 60 °C for 30 s, and 72 °C for 30 s; followed by melting curve analysis from 72 to 95 °C. Gene expression was normalized to *Ubiquitin 5* (Os01g0328400) [63]. Primer sequences are provided in Table S2.

### 4.7. GUS Staining Assay

Genomic DNA was extracted from 21-day-old seedlings as described by [64]. A 1.5 kb upstream promoter region of each TF gene was amplified from 5 ng of genomic DNA and cloned into the pCAMBIA1201 vector. Constructs were introduced into the *Agrobacterium tumefaciens* strain EHA101 and subsequently transformed into embryogenic rice calli derived from mature seeds [65]. Immature seeds at various stages were harvested from transgenic lines for GUS staining, which was conducted following [66] to minimize false negatives. Samples were pretreated in ice-cold 90% acetone (5–15 min), then incubated in staining buffer [50 mM sodium phosphate buffer (pH 7.2), 2 mM each of K_4_[Fe(CN)_6_] and K_3_[Fe(CN)_6_], 2% Triton X-100, 2 mM X-Gluc] under vacuum for 5 min, and then incubated overnight at 37 °C in the dark. Samples were rinsed in 70% ethanol and stored at 4 °C.

### 4.8. Yeast Two-Hybrid Assay

The interactions between TFs were examined using the ProQuest™ Two-Hybrid System with Gateway Technology (Invitrogen, Cat. #PQ1000101, Carlsbad, CA, USA). Full-length cDNAs of each TF were cloned into the bait (pDEST32) and prey (pDEST22) vectors via Gateway cloning. Cloning primers are listed in Table S2. Yeast strain MaV203 was used for transformation and interaction screening.

### 4.9. Cis-Regulatory Element (CRE) Frequency Analysis

The 2000 bp promoter regions upstream of the start codon for 55,802 annotated rice genes (MSU RGAP; http://rice.uga.edu, accessed on 7 August 2025) were extracted and screened for cis-regulatory elements using the PLACE database (https://www.dna.affrc.go.jp/PLACE, accessed on 7 August 2025) via Find Individual Motif Occurrence (FIMO) in the MEME Suite (https://meme-suite.org, accessed on 7 August 2025). Frequencies of individual CREs were computed genome-wide and specifically for nine selected TF promoters. Enrichment was expressed as log_2_ fold change (log_2_FC), and statistical significance was determined using a normal distribution model based on genome-wide CRE occurrence.

## 5. Conclusions

In this study, we identified nine TFs that are co-expressed with major SSP genes in rice through transcriptome-based co-expression network analysis. These TFs—OsGATA8, OsMIF1, OsMIF2, OsGZF1, OsbZIP58, OsS1Fa1, OsS1Fa2, OsICE2, and OsMYB24—exhibited seed-preferential expression patterns, with peak expression during early seed development. Promoter analysis revealed significant enrichment of seed-specific and hormone-responsive CREs, supporting their potential roles in SSP transcriptional regulation. Functional domain prediction, protein–protein interaction analysis, and GUS reporter assays further supported the relevance of these TFs to seed development and nutrient storage. Collectively, these findings provide valuable insights into the transcriptional networks governing rice SSP biosynthesis and offer molecular targets for genome editing to enhance seed quality and nutrient content in rice.

## Figures and Tables

**Table 1:** Information of TFs selected by correlation analysis.

Superclass	Class	Gene Name	MSU ID ^a^	RAP Locus ID ^b^	Functional Description	Reference
Zinc finger	GATA type	OsGATA8	LOC_Os01g24070	Os01g0343300	Response salinity, drought stress, ABA	[21]
	ZF-HD type	OsMIF1	LOC_Os11g03420	Os11g0128300		
		OsMIF2	LOC_Os12g03110	Os12g0124500		
	C3H type	OsGZF1	LOC_Os07g47240	Os07g0668600	Binding with the GluB-1 promoter and controls glutelin content	[18]
Basic domains	Basic leucine zipper factors (bZIP)	OsbZIP58, RISBZ1	LOC_Os07g08420	Os07g0182000	Regulate seed storage protein (SSP) and starch synthesis genes during grain filling stage	[16,17]
	Basic helix–loop–helix factors (bHLH)	OsbHLH001, OsICE2	LOC_Os01g70310	Os01g0928000	Response cold stress and regulate stomatal development	[22,23]
S1Fa	S1Fa	OsS1Fa1	LOC_Os04g33420	Os04g0408900	Response drought stress	[24]
		OsS1Fa2	LOC_Os04g33440	Os04g0408700		
MYB	R2R3 type	OsMYB24	LOC_Os01g74590	Os01g0977300		

^a^ Rice Genome Annotation Project (http://rice.uga.edu/, accessed on 7 August 2025). ^b^ The Rice Annotation Project (RAP, https://rapdb.dna.affrc.go.jp/, accessed on 7 August 2025).

**Table 2:** Enriched cis-regulatory elements in promoters of selected transcription factor genes.

NO.	Gene	Cis element	Consensus Sequence (5′-3′)	Description	Reference
1	*OsMYB24*	ABREZMRAB28	CCACGTGG	ABA and water–stress response element	[25]
2	*OsMYB24*	O2F2BE2S1	GCCACCTCAT	Seed-specific expression element	[26]
3	*OsMYB24*	RYREPEATGMGY2	CATGCAT	Seed-specific expression element	[27]
4	*OsMYB24*	RYREPEATVFLEB4	CATGCATG	ABA response and Seed storage protein expression element	[28]
5	*OsbZIP58*	AMYBOX1	TAACARA	Seed-specific expression element	[29]
6	*OsbZIP58*	AUXRETGA2GMGH3	TGACGTGGC	Auxin-responsive element	[30]
7	*OsbZIP58*	GARE1OSREP1	TAACAGA	Seed-specific expression and GA-responsive element	[31]
8	*OsGATA8*	-300ELEMENT	TGHAAARK	Seed storage protein expression element	[32]
9	*OsGZF1*	SEF4MOTIFGM7S	RTTTTTR	Seed storage protein expression element	[33]
10	*OsICE2*	TATABOX1	CTATAAATAC	TATA box-like and Seed-specific expression element	[34]

## Data Availability

All data generated and analyzed during this study are included in this published article and its additional files.

## Supplementary Materials

The following supporting information can be downloaded at: https://www.mdpi.com/article/10.3390/plants14172791/s1. Figure S1. Transient over-expression of candidate TFs in rice protoplasts and their effects on seed storage protein (SSP) gene expression.; Figure S2. Interaction between TFs in yeast two-hybrid assays.; Figure S3. Confirmation of *pTF::GUS* transgenic rice.; Figure S4. Volcano plot of cis-element enrichment in TF promoters.; Table S1. Pearson’s correlation values between genes assigned to transcription factor and seed storage proteins.; Table S2. Primer list; Table S3. Proteins interacting with the selected transcription factors; Table S4. Annotation of proteins interacting with the selected transcription factors; Table S5. Cis-regulatory elements in the 2000 bp region upstream of the translation start codon of selected TF genes in rice identified through genome-wide frequency comparison; Table S6. Cis-regulatory element localization within promoter regions of selected TF genes.

## Abbreviations

The following abbreviations are used in this manuscript:

ABA	Abscisic acid
bHLH	Basic helix–loop–helix
bZIP	Basic leucine zipper
CRE	Cis-regulatory element
CRISPR-Cas9	Clustered regularly interspaced short palindromic repeats–CRISPR associated protein 9
DAF	Days after flowering
FIMO	Find Individual Motif Occurrence
GATA	GATA-type zinc finger protein
GEO	Gene Expression Omnibus
GUS	β-glucuronidase
MIF	Mini zinc finger protein
MSU	Michigan State University
MYB	Myeloblastosis-related transcription factor
PPI	Protein–protein interaction
PLACE	Plant cis-acting regulatory DNA elements database
RAP-DB	Rice Annotation Project Database
RGAP	Rice Genome Annotation Project
RNAi	RNA interference
RPBF	Rice Prolamin Box Binding Factor
S1F	Site 1 Factor
SSP	Seed storage proteins
STRING	Search Tool for the Retrieval of Interacting Genes/Proteins
TF	Transcription factor
Y2H	Yeast two-hybrid
ZF-HD	Zinc finger homeodomain
