# Supplementary material for: Transcriptome-Based Identification of Novel Transcription Factors Regulating Seed Storage Proteins in Rice
Source: Plants (Basel). 2025 Sep 5;14(17):2791. doi: 10.3390/plants14172791 (PMC12431501; doi:10.3390/plants14172791)
Supplement: Supplementary file 1 [file plants-14-02791-s001.zip › Supplementary Materials.pdf]

Transcriptome-Based Identification of Novel Transcription Factors Regulating Seed Storage Proteins in Rice

Supplementary materials

Figure S1. Transient over-expression of candidate TFs in rice protoplasts and their effects on seed storage protein (SSP) gene expression.

Figure S2. Interaction between TFs in yeast two-hybrid assays.

Figure S3. Confirmation of *pTF::GUS* transgenic rice.

Figure S4. Volcano plot of cis-element enrichment in TF promoters.

Table S1. Pearson's correlation values between genes assigned to transcription factor and seed storage proteins.

Table S2. Primer list

Table S3. Proteins interacting with the selected transcription factors

Table S4. Annotation of proteins interacting with the selected transcription factors

Table S5. Cis-regulatory elements in the 2,000 bp region upstream of the translation start codon of selected TF genes in rice identified through genome-wide frequency comparison

Table S6. Cis-regulatory element localization within promoter regions of selected TF genes

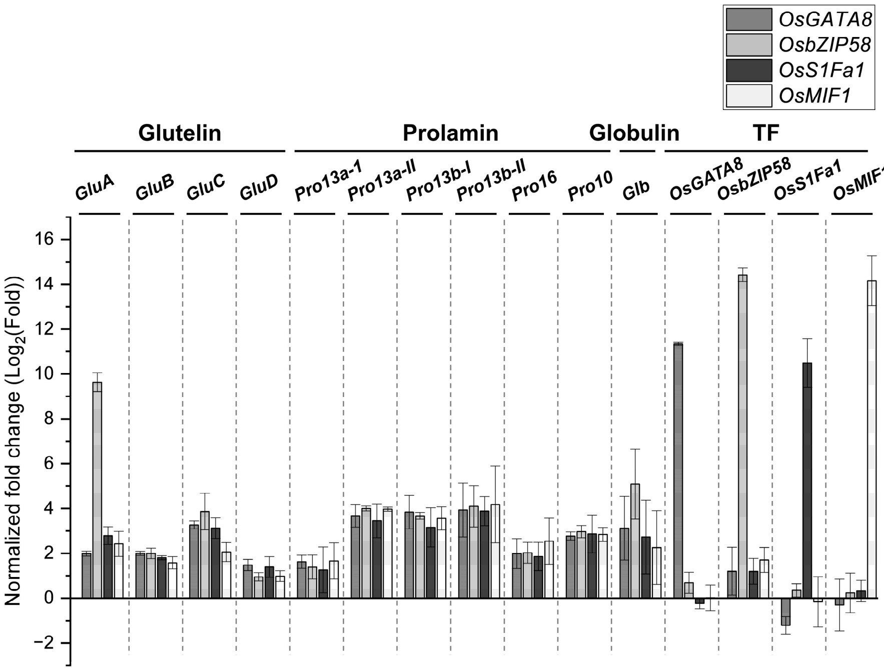

Figure S1. Transient overexpression of candidate TFs in rice protoplasts and their effects on seed storage protein (SSP) gene expression. Rice protoplasts were transfected with plasmids carrying *OsGATA8*, *OsbZIP58*, *OsS1Fa1*, or *OsMIF1*, and transcript levels of SSP genes were determined by qRT-PCR. Normalization of expression levels of the target genes was performed using the $2^{-\Delta\Delta CT}$ method and the relative level of each gene in TF-overexpressing protoplasts versus control is represented as $\log_2$ (average fold change) value. *OsUBI10* was used as the internal control. Values are mean ± SD (n = 3).

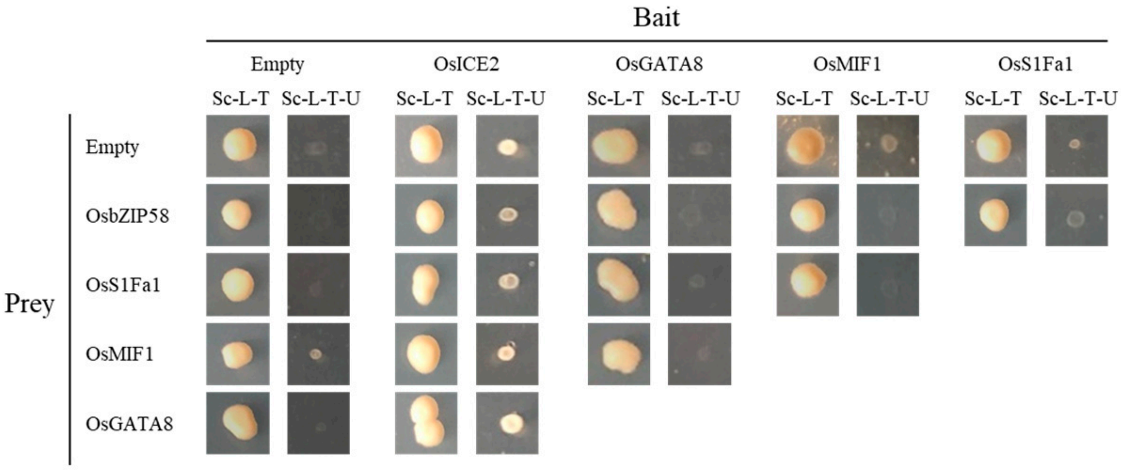

Figure S2. Interaction between TFs in yeast two-hybrid assays. The co-transformed strains were spotted on SC-L-T/SC-L-T-U plates to test the interaction between the expressed proteins. The empty pDEST32 (bait) and pDEST22 (prey) vectors were used as negative controls. Sc; Synthetic Complete, L; Leu, T; Trp, U; Ura.

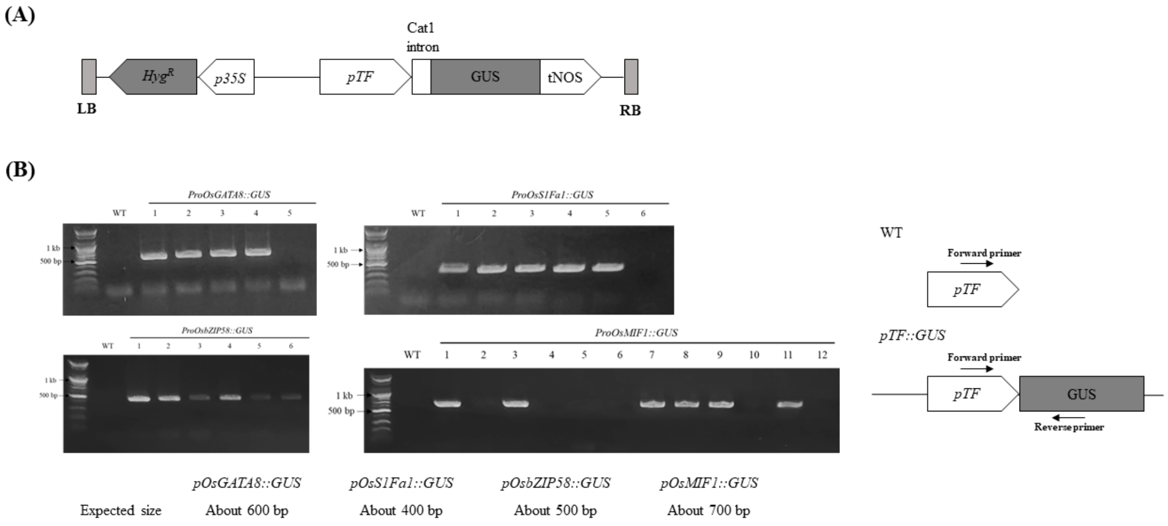

Figure S3. Confirmation of *pTF::GUS* transgenic rice. **(A)** Schematic diagram of TF promoter-GUS expression vector. **(B)** PCR analysis of the T_1 transgenic rice showing the amplification of GUS gene with TF promoter.

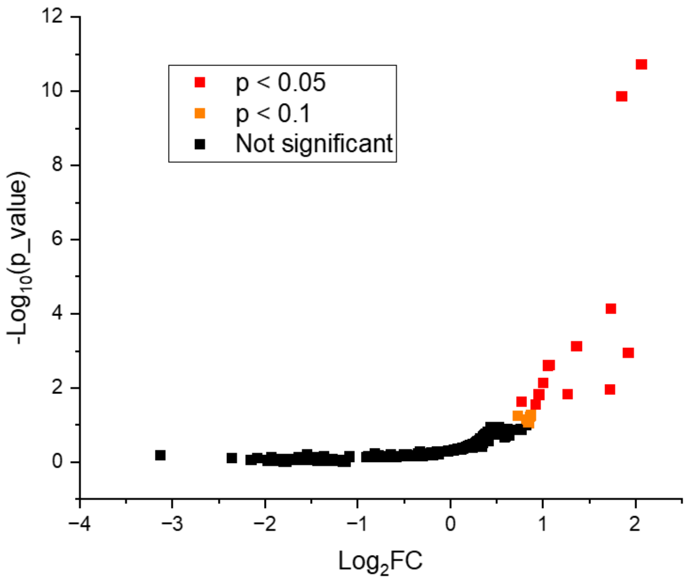

Figure S4. Volcano plot of cis-element enrichment in TF promoters. Volcano plot showing the differential distribution of cis-regulatory elements based on average motif frequencies across the genome compared to the promoter regions of selected transcription factor genes. Each point represents a cis-element associated with one of the selected TF genes. The x-axis indicates the $\log_2$ fold change ($\log_2$FC) in motif frequency between the TF gene set and the genome-wide average, while the y-axis shows the $-\log_{10}$ transformed p-value derived from a normal distribution based on background motif frequency. Red squares represent cis-elements with p-values < 0.05, orange squares indicate $0.05 \leq p < 0.1$, and black squares denote $p \geq 0.1$.
