# Supplementary figures and images for: Transcriptome-Based Identification of Novel Transcription Factors Regulating Seed Storage Proteins in Rice
Source: Plants (Basel). 2025 Sep 5;14(17):2791. doi: 10.3390/plants14172791 (PMC12431501; doi:10.3390/plants14172791)

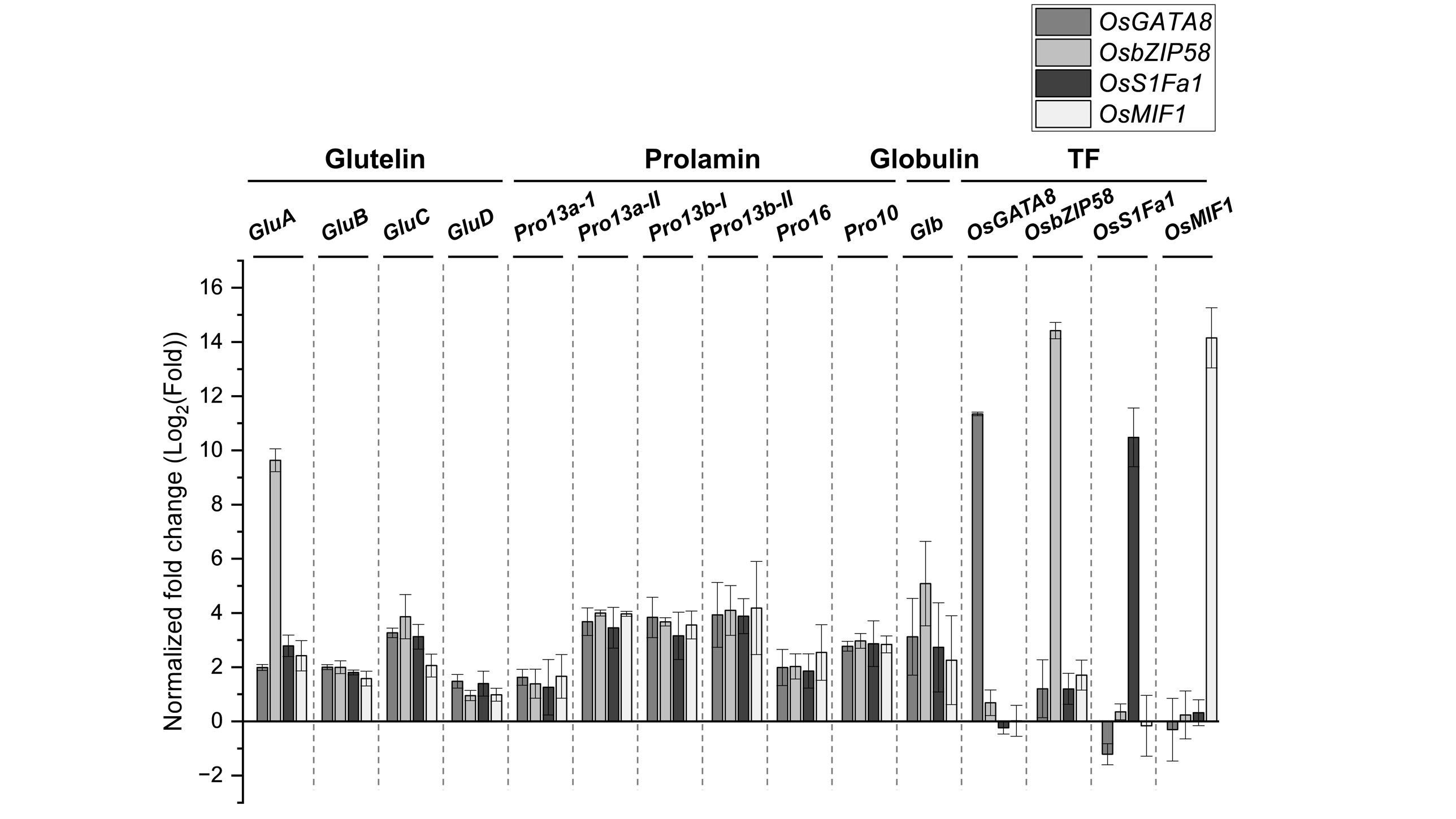

Supplement: Supplementary file 1 [file plants-14-02791-s001.zip › Figure S1.tif]

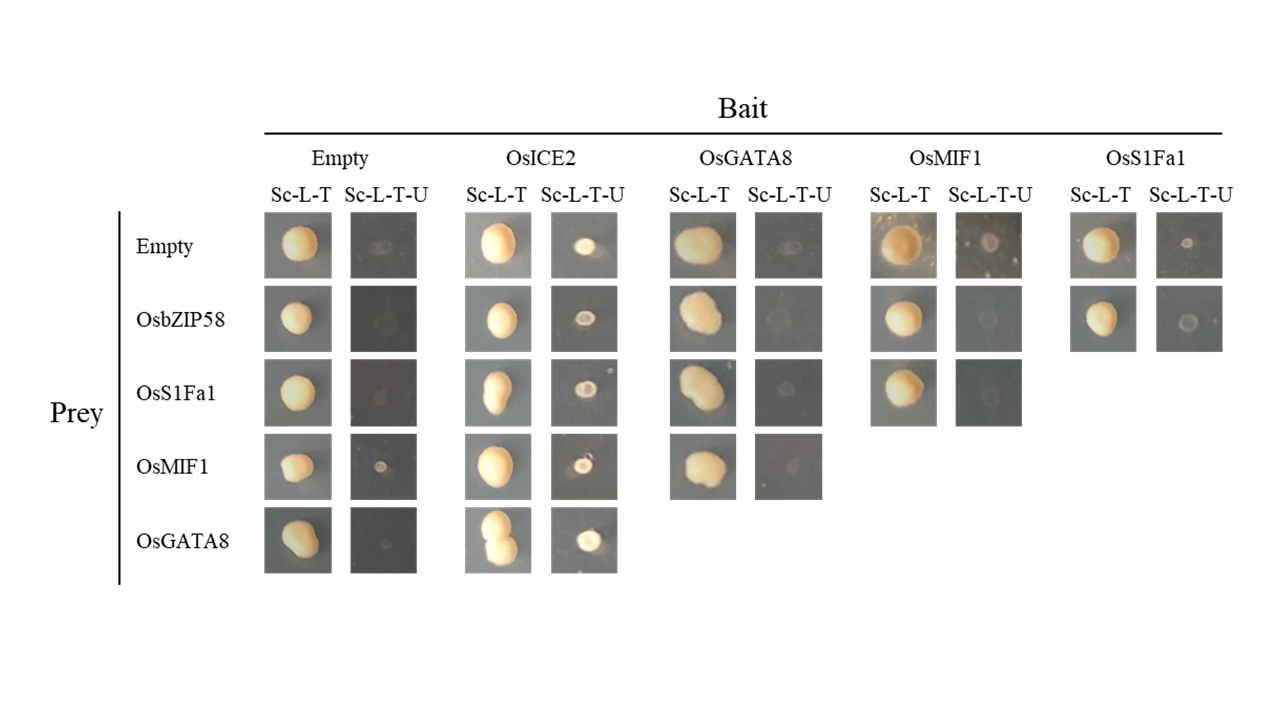

Supplement: Supplementary file 1 [file plants-14-02791-s001.zip › Figure S2.TIF]

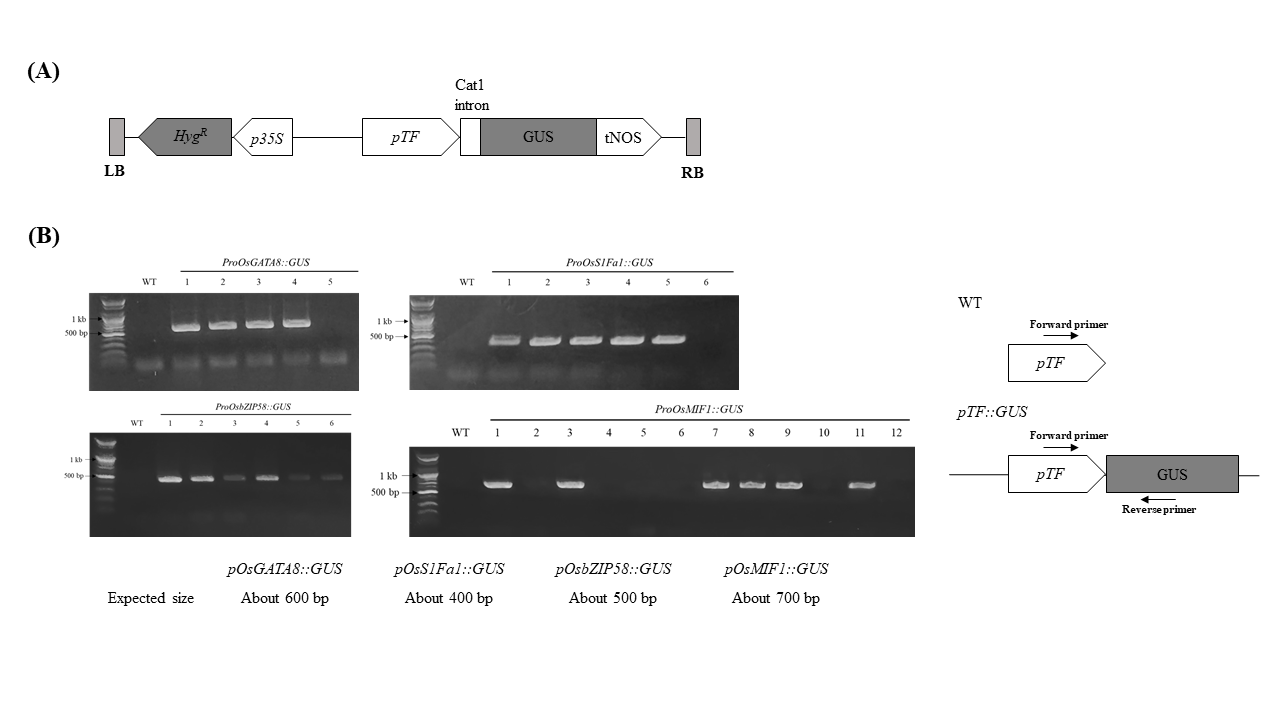

Supplement: Supplementary file 1 [file plants-14-02791-s001.zip › Figure S3.TIF]

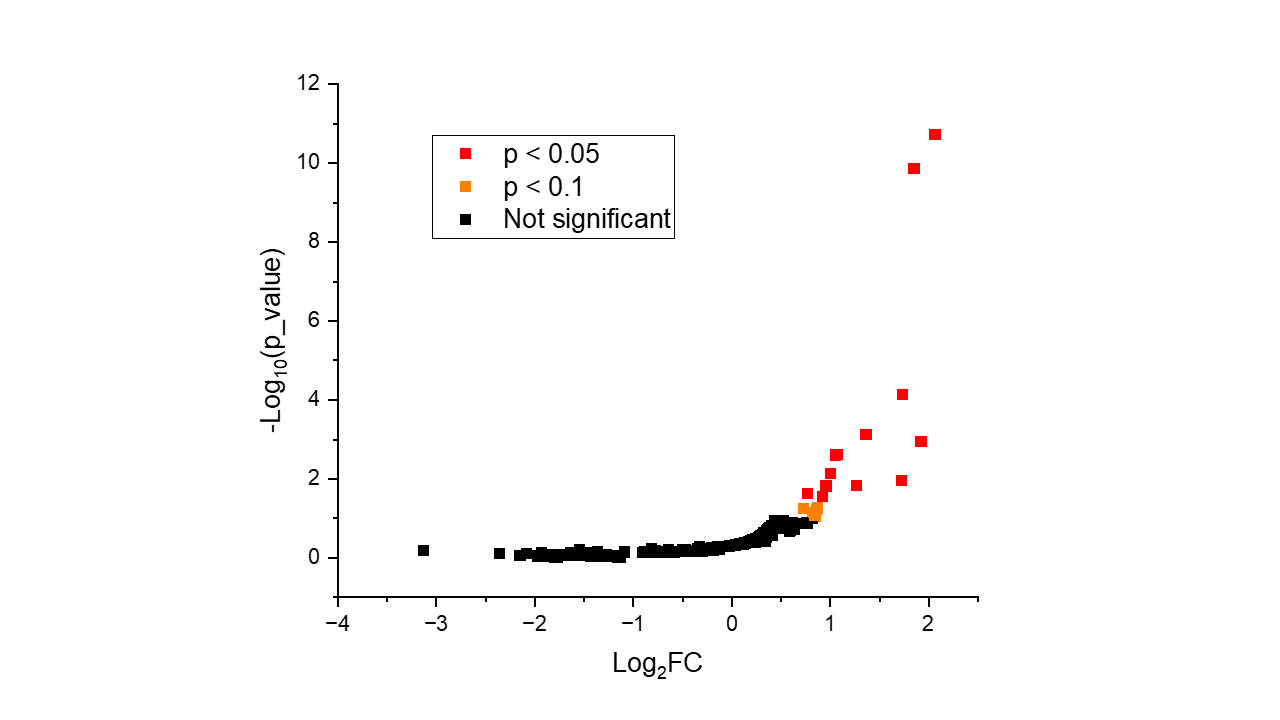

Supplement: Supplementary file 1 [file plants-14-02791-s001.zip › Figure S4.TIF]
